# Fluorescence and Magnetic Resonance Dual-Modality Imaging-Guided Photothermal and Photodynamic Dual-Therapy with Magnetic Porphyrin-Metal Organic Framework Nanocomposites

**DOI:** 10.1038/srep44153

**Published:** 2017-03-08

**Authors:** Hui Zhang, Yu-Hao Li, Yang Chen, Man-Man Wang, Xue-Sheng Wang, Xue-Bo Yin

**Affiliations:** 1 State Key Laboratory of Medicinal Chemical Biology and Tianjin Key Laboratory of Biosensing and Molecular Recognition, College of Chemistry, Nankai University, Tianjin 300071, China; 2 Tianjin Key Laboratory of Tumor Microenviroment and Neurovascular Regulation, School of Medicine, Nankai University, Tianjin 300071, China; 3 School of Public Health, North China University of Science and Technology, Tangshan 063000, Hebei, China; 4 Collaborative Innovation Center of Chemical Science and Engineering (Tianjin), Nankai University, Tianjin 300071, China

## Abstract

Phototherapy shows some unique advantages in clinical application, such as remote controllability, improved selectivity, and low bio-toxicity, than chemotherapy. In order to improve the safety and therapeutic efficacy, imaging-guided therapy seems particularly important because it integrates visible information to speculate the distribution and metabolism of the probe. Here we prepare biocompatible core-shell nanocomposites for dual-modality imaging-guided photothermal and photodynamic dual-therapy by the *in situ* growth of porphyrin-metal organic framework (PMOF) on Fe_3_O_4_@C core. Fe_3_O_4_@C core was used as *T*_*2*_-weighted magnetic resonance (MR) imaging and photothermal therapy (PTT) agent. The optical properties of porphyrin were well remained in PMOF, and PMOF was therefore selected for photodynamic therapy (PDT) and fluorescence imaging. Fluorescence and MR dual-modality imaging-guided PTT and PDT dual-therapy was confirmed with tumour-bearing mice as model. The high tumour accumulation of Fe_3_O_4_@C@PMOF and controllable light excitation at the tumour site achieved efficient cancer therapy, but low toxicity was observed to the normal tissues. The results demonstrated that Fe_3_O_4_@C@PMOF was a promising dual-imaging guided PTT and PDT dual-therapy platform for tumour diagnosis and treatment with low cytotoxicity and negligible *in vivo* toxicity.

Cancer becomes one of the major threats to human beings although great efforts have been done^1^. Chemotherapy is still the popular means for cancer therapy, but drug-resistance and bio-toxicity are its important limitations^2^. As a consequence, various physiotherapy strategies emerge at the right moment. Phototherapy, including photothermal therapy (PTT) and photodynamic therapy (PDT), shows unique advantages, such as remote controllability, improved selectivity, and low bio-toxicity compared with chemotherapy^3^,^4^,^5^. Probing the position of photoactive agent becomes important for phototherapy, so multifunctional cancer therapy platforms (MCTPs) attract much attention as they integrate imaging and therapy into a single system for imaging-guided therapy to improve the therapeutic efficiency and safety^6^,^7^. Various interesting MCTPs have been fabricated with emissive nanostructures (e.g., Au nanorods and nanoshells) and photosensitizers (e.g., chlorine e6 and indocyanine green) for imaging-synergistic-therapy^8^,^9^. However, the systems suffered from single-modality imaging, single-therapy, and/or complex post-modification. If multi-modality imaging is selected, the advantages of each imaging modality are integrated together, such as high sensitivity of fluorescence and the deep penetration and spatial resolution of magnetic resonance (MR) imaging^10^,^11^,^12^. The dual-therapy combination of PTT and PDT could improve the therapeutic effect significantly 13,14,15.

One of the challenges to build MCTPs is the selection of safe and biocompatible building blocks with optical and/or magnetic responses. Porphyrin and its derivatives are widely used as photosensitizers and organic ligands for bioimaging and PDT^16^, due to their unique optoelectronic properties^17^,^18^. However, their large hydrophobic planar structure makes porphyrin easily aggregated to quench their fluorescence and decrease the capacity of singlet oxygen generation^19^. Porphyrin-metal-organic frameworks (PMOFs) have the rigid structure and well retain the optoelectronic property of porphyrin.

Superparamagnetic iron oxide nanoparticles (SPIONs) are efficient imaging agents because they shorten transverse relaxation with facile synthesis and excellent biocompatibility^20^,^21^. The cluster structure of Fe_3_O_4_ nanoparticles is effective to enhance MR imaging efficiency than single-domain nanocrystals because it impairs the longitudinal relaxivity^20^,^21^. Moreover, the number and magnetic moment of nanoparticles in an assembly are proportional to transverse relaxivity (*r*_*2*_)^20^,^21^. Thus, selection of simple Fe_3_O_4_ cluster preparation is useful to improve *T*_*2*_-MR imaging efficiency^19^. SPIONs are biodegradable^22^ and ferumoxsil and ferumoxide, therefore have been approved by Food and Drug Administration as MR contrast agents^23^,^24^,^25^. Fe_3_O_4_ nanoparticles can convert near-infrared (NIR) irradiation to heat to enable localized damage to cancer cells or tissues. Thus, the combination of magnetic property and local photothermal effect make SPIONs interesting as imaging and therapy agents.

Herein, we report the fluorescence-MR dual-modality imaging-guided PTT-PDT dual-therapy system with novel core-shell Fe_3_O_4_@C@PMOF nanocomposites. A cluster of Fe_3_O_4_ nanoparticles were encapsulated in the carbon shell as Fe_3_O_4_@C for *T*_*2*_-weighted MR imaging and as PTT agent. PMOF was then post-modified on the biocompatible and stable Fe_3_O_4_@C^26^,^27^. The PMOF was constructed with highly biocompatible and stable Zr ions and TCPP [5, 10, 15, 20–Tetrakis (4-carboxyl)-21H, 23H-porphine] as fluorescence imaging and PDT agent^17^,^28^,^29^,^30^. Biocompatible core-shell Fe_3_O_4_@C@PMOF nanocomposites were therefore fabricated for *T*_*2*_-weighted MR and fluorescence dual-modality imaging-guided PTT and PDT dual-therapy. Tumor-bearing mice experiment demonstrated the high tumor accumulation of Fe_3_O_4_@C@PMOF as efficient MCTP after irradiated with lasers. Low cytotoxicity and bio-toxicity of Fe_3_O_4_@C@PMOF were validated as the high biocompatibility of the building blocks. To our knowledge, this is the first Fe_3_O_4_@C@PMOF reported as MCTPs for dual-modality imaging-guided dual-therapy and realized the cancer therapy without chemical drugs.

## Results

### Synthesis and Characterization of Fe_3_O_4_@C and Fe_3_O_4_@C@PMOF

**Figure 1:**
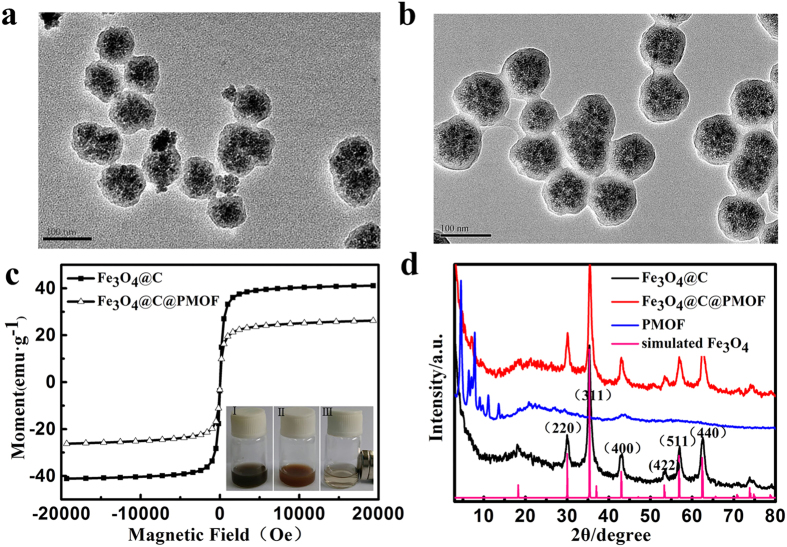
TEM images, magnetic hysteresis curves, and XRD patterns of nanoparticles. TEM images of (**a**) Fe_3_O_4_@C and (**b**) Fe_3_O_4_@C@PMOF; (**c**) Magnetic hysteresis curves of Fe_3_O_4_@C and Fe_3_O_4_@C@PMOF samples at 300 K. Inset: photo images of (I) Fe_3_O_4_@C, (II) Fe_3_O_4_@C@PMOF, and (III) magnetic collection of Fe_3_O_4_@C@PMOF; (**d**) XRD patterns of synthesized Fe_3_O_4_@C@PMOF, PMOF, Fe_3_O_4_@C, and simulated Fe_3_O_4_.

Fe_3_O_4_@C micro-structure was fabricated by one-pot solvothermal strategy according to previous report^31^. Fe_3_O_4_@C nanoparticles were dispersed in the DMF suspension of ZrCl_4_ in a hydrothermal procedure for 30 min, and then DMF solution of TCPP was added into the mixture. Fe_3_O_4_@C@PMOF was prepared by the *in situ* self-assembly of PMOF on the surface of Fe_3_O_4_@C to obtain the core-shell nanocomposites. The proposed method was time-saving and efficient compared with the layer-by-layer self-assembly of MOF^32^,^33^. Therefore, a simple strategy was developed to prepare the Fe_3_O_4_@C@PMOF composite. Transmission electron microscopy (TEM) images of Fe_3_O_4_@C and Fe_3_O_4_@C@PMOF revealed their well-defined micro-structure with the average diameter of 80 and 95 nm, respectively (Fig. 1a and b). Moreover, ca 7.5 nm PMOF layer was successfully coated on Fe_3_O_4_@C to form the Fe_3_O_4_@C@PMOF hybrid nanocomposites. Fe_3_O_4_@C nanoclusters consisted of numerous 10 nm Fe_3_O_4_ nanoparticles as illustrated in Fig. 1a and b, different to the solid Fe_3_O_4_ structure of ferumoxsil and ferumoxide^23^,^24^,^25^. Thus, improved *T*_*2*_-MR imaging efficiency is expected because of the altered proton relaxation effect of the nanocluster structure. The composites less than 100 nm pass through the tumor microenvironment easily and remain for a long time before blood clearance^34^. Moreover, the size of Fe_3_O_4_@C@PMOF was suitable for PDT because the diffusion length of singlet oxygen (^1^O_2_) was 90−120 nm in aqueous environment and 20−220 nm inside cells^28^.

Dynamic light scattering analysis indicated that Fe_3_O_4_@C@PMOF had a relatively narrow size distribution and was well dispersed for real application (Supplementary Fig. S1). −15.7 and −3.39 mV of zeta potentials were observed for Fe_3_O_4_@C and Fe_3_O_4_@C@PMOF (Supplementary Fig. S2). Therefore, the carbon layer of Fe_3_O_4_@C was oxidized by H_2_O_2_ under solvothermal procedure to form abundant carboxylic groups, which were then used to coordinate with Zr^4+^ ions. The PMOF layer formed through the coordination between Zr^4+^ ions and TCPP as the zeta potential changed from original −15.7 mV to −3.39 mV. The near-neutral surface of the nanocomposites makes them excellent candidates for *in vivo* applications^35^.

The magnetic properties of Fe_3_O_4_@C and Fe_3_O_4_@C@PMOF were characterized by Vibrating Sample Magnetometer (VSM) at the field of ± 20 kOe (Fig. 1c). The saturation magnetization of Fe_3_O_4_@C was 39.8 emu g^−1^. The magnetic hysteresis curve was retained in Fe_3_O_4_@C@PMOF with the saturation magnetization of 24.5 emu g^−1^. Both Fe_3_O_4_@C and Fe_3_O_4_@C@PMOF were well dispersed, but they were collected easily with external magnet and the solution became transparent (Inset in Fig. 1c). Thus, the great MR imaging potential was revealed from Fe_3_O_4_@C@PMOF.

Powder X-ray diffraction (XRD) patterns of Fe_3_O_4_@C, PMOF, and Fe_3_O_4_@C@PMOF were recorded (Fig. 1d). The peaks observed at 30.1, 35.3, 42.9, 53.5, 57.0, and 62.5° were assigned to (220), (311), (400), (422), (511) and (440) planes of cubic structure of Fe_3_O_4_ crystal (JCPDS No.75-1609). The simultaneous existence of the characteristic peaks of Fe_3_O_4_ and PMOF in its XRD pattern indicates the successful formation of Fe_3_O_4_@C@PMOF nanocomposites. The formation was also confirmed by Fourier transform infrared spectroscopy (FT-IR) with the characteristic peak at 964.45 cm^−1^ assigned to the pyrrole ring of the ligand, TCPP (Supplementary Fig. S3). Thermogravimetric analysis (TGA) results revealed that Fe_3_O_4_@C was highly stable in the tested temperature (Supplementary Fig. S4). The gradual weight loss before 200 °C was attributed to the removal of solvents, including acetone and DMF, from both Fe_3_O_4_@C and Fe_3_O_4_@C@PMOF. The removal of carbon shell of Fe_3_O_4_@C was at around 300 °C. The large weight loss of Fe_3_O_4_@C@PMOF occurred at around 400 °C was assigned to the collapse of the PMOF skeleton upon the decomposition of TCPP.

### Optical properties and MR response of Fe_3_O_4_@C@PMOF

**Figure 2:**
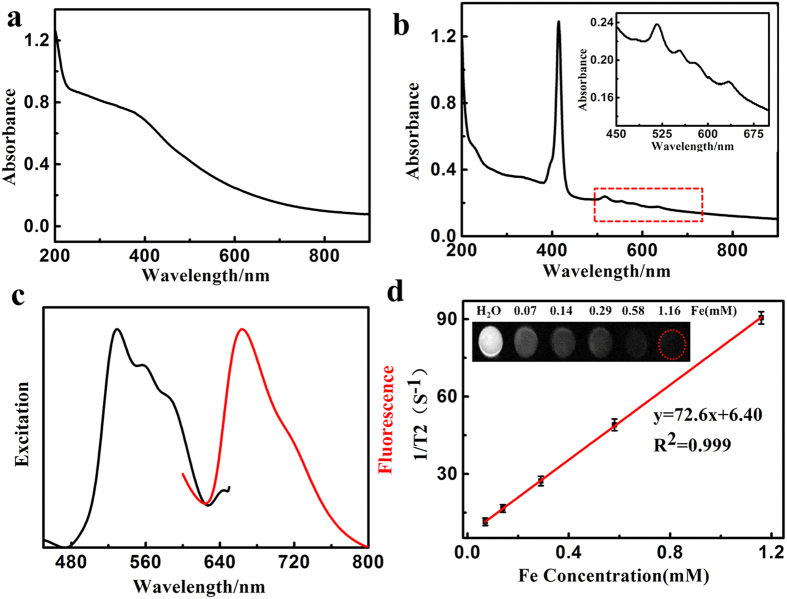
Optical properties and MR response of Fe_3_O_4_@C@PMOF. UV−Vis spectra of (**a**) Fe_3_O_4_@C and (**b**) Fe_3_O_4_@C@PMOF nanocomposites. Inset: the amplification of the part spectrum. (**c**) The excitation and fluorescence spectra (excitation at 553 nm) of Fe_3_O_4_@C@PMOF aqueous solution. (**d**) *T*_*2*_-weighted MR images of Fe_3_O_4_@C@PMOF at different concentrations of Fe and the plot of 1/*T*_*2*_ over Fe concentration of Fe_3_O_4_@C@PMOF nanocomposites. The slope indicates the specific relaxivity (*r*_*2*_).

The UV–Vis spectra of Fe_3_O_4_@C and Fe_3_O_4_@C@PMOF dispersed in aqueous solution were recorded. An extended absorption band was observed in NIR region from Fe_3_O_4_@C (Fig. 2a). This feature provided efficient photothermal capacity. When PMOF shell was covered, a strong absorption peak emerged at 416 nm for Soret band (Fig. 2b) and four peaks at 517, 554, 583, and 634 nm were observed for Q band as the typical character of porphyrin (inset of Fig. 2b)^16^. Thus, Fe_3_O_4_@C@PMOF was potential for PDT because of its matched NIR absorption^36^. Single emission peak was observed at 668 nm from Fe_3_O_4_@C@PMOF with 553 nm excitation (Fig. 2c). Strong NIR emission and long Stocks shift led to a high signal/noise ratio for fluorescent image because of the low auto-fluorescence and scattering light from biological tissue^37^,^38^. The optical property of Fe_3_O_4_@C@PMOF illustrated its PTT and PDT potential and fluorescence imaging capacity.

*T*_*2*_-weighted imaging was tested to validate the MR contrast potential of Fe_3_O_4_@C@PMOF nanocomposites. The relaxation rates vary linearly with increased Fe concentration (Fig. 2d). The darkening *T*_*2*_ MR imaging at different Fe concentrations confirmed the *T*_*2*_-weighted MR efficiency (Inset of Fig. 2d). Fe content of Fe_3_O_4_@C@PMOF was tested by inductively coupled plasma-atomic emission spectroscopy, and the slope in Fig. 2d was the *r*_*2*_ value, which was 72.6 mM^−1 ^s^−1^ (*r*_*1*_ = 1.23 mM^−1 ^s^−1^, *r*_*2*_/*r*_*1*_ = 59.0, Supplementary Fig. S5). The *r*_*2*_ value was higher than that of commercial magnetic nanoparticles (10 nm, *r*_*2*_ = 59.91 ± 6 mM^−1 ^s^−1^)^39^ due to the integration of numerous 10 nm Fe_3_O_4_ nanoparticles in a carbon layer for efficient *T*_*2*_*-*contrast effect^21^. The *r*_*2*_/*r*_*1*_ ratio was 59.0, and therefore Fe_3_O_4_@C@PMOF showed the potential for *T*_*2*_-weighted MR imaging.

### I*n vitro* photothermal and photodynamic properties of Fe_3_O_4_@C@PMOF.

**Figure 3:**
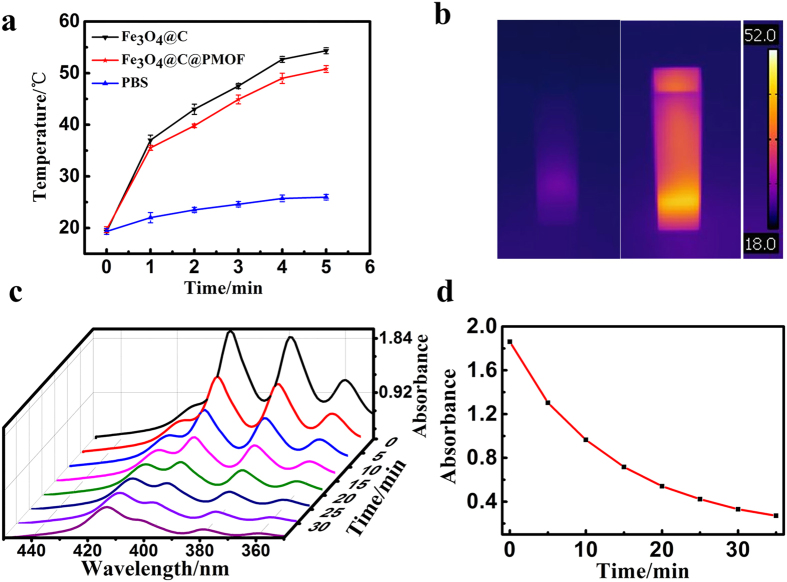
Photothermal and photodynamic properties of Fe_3_O_4_@C@PMOF. (**a**) Temperature of Fe_3_O_4_@C, Fe_3_O_4_@C@PMOF, and PBS solutions as a function of time upon exposure to 808 nm laser at 1.0 W cm^−2^ within 5 min; (**b**) Infrared thermal photograph of Fe_3_O_4_@C@PMOF solution before and after 808 nm irradiation (1.0 W cm^-2^) for 5 min. (**c**) Absorbance spectra of ABDA (200 μmol L^−1^) in the presence of Fe_3_O_4_@C@PMOF nanocomposites (20 μmol L^−1^) over different periods under irradiation of 655 nm (0.3 W cm^−2^) in pH 7.4 PBS solution. (**d**) Absorbance values of ABDA at 379 nm against irradiation time in the presence of Fe_3_O_4_@C@PMOF nanocomposites.

PTT efficiency of Fe_3_O_4_@C@PMOF was evaluated by measuring the temperature change under 808 nm NIR laser irradiation (Fig. 3a). After a 5 min of laser exposure, the solutions containing Fe_3_O_4_@C and Fe_3_O_4_@C@PMOF respectively were rapidly heated to higher than 50 °C because of the high NIR absorptivity of Fe_3_O_4_, while the PBS solution showed less photo-generated heating efficiency (Supplementary Fig. S6). However, when Fe_3_O_4_@C@PMOF solution was exposed to 655 nm laser for 30 min, the temperature gave rise to 29 °C, which was negligible to damage cancer cells (Supplementary Fig. S7). Infrared thermal photographs of Fe_3_O_4_@C@PMOF solution before and after 808 nm irradiation illustrated the photothermal capacity directly (Fig. 3b).

Singlet oxygen (^1^O_2_) is the electronic excited state of molecular oxygen and highly reactive in the oxidization damage of biological tissues^4^,^28^. 9, 10-anthracenediyl-bis (methylene) dimalonic acid (ABDA) was used as indicator to verify the ^1^O_2_ generation capacity of Fe_3_O_4_@C@PMOF because ABDA can react with ^1^O_2_ irreversibly^40^. The reaction was monitored by the decreased ABDA absorption at 379 nm. The absorption spectra of ABDA in Fe_3_O_4_@C@PMOF were recorded at different exposure times (Fig. 3c), and the variation of absorbance was illustrated in Fig. 3d. The rapid decrease of ABDA absorption represented fast ^1^O_2_ generation by Fe_3_O_4_@C@PMOF under 655 nm laser irradiation. However, ABDA was stable for single Fe_3_O_4_@C@PMOF or light (Supplementary Fig. S8). The ^1^O_2_ generation rate was calculated with the following equation^13^,^28^,^41^:


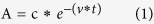


where *A* refers to the absorption of ABDA at 379 nm, *c* is fitting parameter, and *t* is irradiation time. The ^1^O_2_ generation rate (*v*) was calculated as 0.055 min^−1^ for Fe_3_O_4_@C@PMOF. The ^1^O_2_ quantum yield is calculated with the equation^40^:


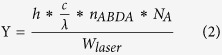


where *h* is Plank constant, *c* is velocity of light, *λ* is the wavelength of laser, *n*_*ABDA*_ is the amount of ABDA consumed by ^1^O_2_, *N*_*A*_ is Avogadro constant, and *W*_*laser*_ is the power of laser irradiated. The ^1^O_2_ quantum yield (*Y*) of Fe_3_O_4_@C@PMOF was 44.38%, illustrating the high efficiency of Fe_3_O_4_@C@PMOF for ^1^O_2_ generation. In the same way, the ABDA absorption peak at 379 nm was stable under 808 nm laser irradiation (Supplementary Fig. S9). Thus, their optical properties were well remained in the nanocomposites. Moreover, PTT and PDT were regarded as two independent processes from Fe_3_O_4_@C and PMOF, respectively.

### Cytotoxicity and photoxicity of Fe_3_O_4_@C@PMOF

**Figure 4:**
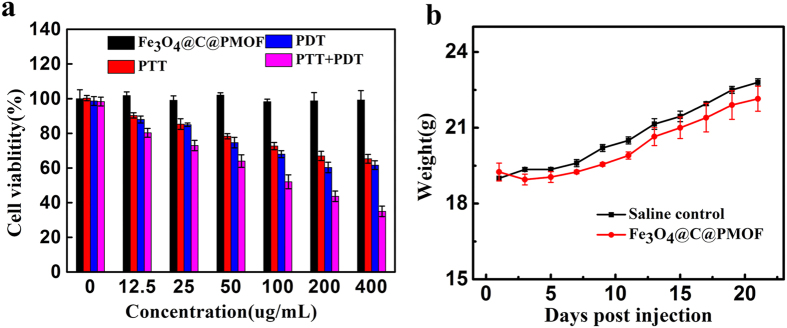
Toxicity of Fe_3_O_4_@C@PMOF. (**a**) Viability of MCF-7 cells incubated with Fe_3_O_4_@C@PMOF at varied concentrations for 8 h with or without laser irradiation. (**b**) The body weight trends of mice within 3 weeks after injection with saline solution (control) or Fe_3_O_4_@C@PMOF (20 mg kg^−1^). Error bars represent the standard deviations of three mice per group.

The cytotoxicity and photoxicity of Fe_3_O_4_@C@PMOF were measured with standard 3-(4, 5-dimethylthiazol-2-yl)−2, 5-diphenyltetrazolium bromide (MTT) assay (Fig. 4a). The cell viability of MCF-7 cells was recorded after incubated with Fe_3_O_4_@C@PMOF at various concentrations for 8 h. The PDT group was irradiated under 655 nm for 10 min, while the PTT group was subjected to 808 nm NIR laser for 10 min. The cell viability of irradiated groups gradually decreased with increasing the concentration of Fe_3_O_4_@C@PMOF. In contrast, the group without irradiation showed negligible cytotoxicity to MCF-7 cells, indicating the high biocompatibility of Fe_3_O_4_@C@PMOF. It is worth noting that viability of cells incubated with Fe_3_O_4_@C@PMOF was remarkably decreased to less than 35% after the co-therapy of PDT and PTT. The long-term biotoxicity of Fe_3_O_4_@C@PMOF was evaluated by monitoring the body weight trend of mice after intravenous injection of Fe_3_O_4_@C@PMOF, and the saline-injected mice was regarded as control. No sign of illness and activity changes was observed from the mice, which also showed the same weight trend to the mice in control group within 3 weeks (Fig. 4b).

### *In vivo* dual-modality imaging of mice with Fe_3_O_4_@C@PMOF as probe and clearance study

**Figure 5:**
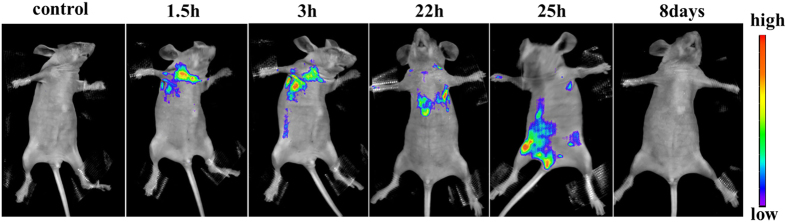
Metabolism processes of Fe_3_O_4_@C@PMOF in nude mice. Fluorescence images of the mice were recorded at excitation of 550 nm and emission of 660 nm under different period after injection of Fe_3_O_4_@C@PMOF. After 8 days, the mice were still alive and well.

To prove the *in vivo* efficiency of dual-modality imaging, Fe_3_O_4_@C@PMOF nanocomposite was injected intravenously into a 20 g healthy nude mouse. *In vivo T*_*2*_ MR image was recorded (Supplementary Fig. S10). The liver region was darkening after being injected for 22 h. The same result was observed in fluorescence imaging simultaneously. The fluorescent spot was also observed in lymph possibly because of the high affinity between porphyrin and lymph node. Thus, the nanocomposites did transfer not only through blood circulation but also participate in the lymph circulation simultaneously. Then, it accumulated in liver. Finally, most of Fe_3_O_4_@C@PMOF was excreted through excrement within 8 days with the similar metabolic pathway to coproporphyrin^42^. The images in Fig. 5 illustrated the whole metabolism process of Fe_3_O_4_@C@PMOF in nude mice after intravenously injected at different time.

**Figure 6:**
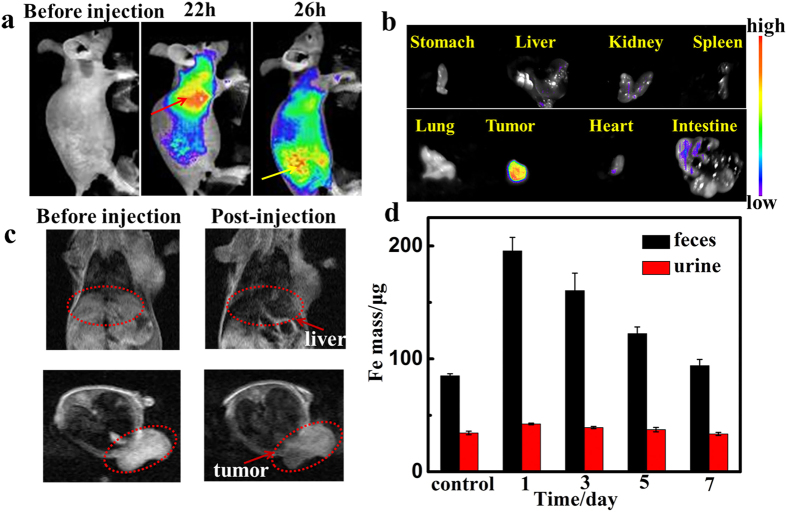
*T*_*2*_-weighted MR and fluorescent imaging of tumor-bearing mice and dissected organs. (**a**) Fluorescent imaging (Ex = 550 nm, Em=660 nm) of tumor-bearing mice before and after intravenous injection of Fe_3_O_4_@C@PMOF (20 mg kg^−1^). The liver region was marked by red dot line and the yellow lines referred to tumor region. (**b**) Fluorescent imaging of dissected organs of tumor-bearing mouse.(**c**) *T*_*2*_-weighted MR imaging of tumor-bearing mice in the coronal plane (upper) and in the axial plane (lower). The liver region and tumor region were marked by red dot line. (**d**) The detected Fe mass in feces and urine at different time points after intravenous injection of Fe_3_O_4_@C@PMOF (20 mg kg^−1^).

To verify the accumulation of Fe_3_O_4_@C@PMOF in tumor site, MCF-7 tumor-bearing nude mice were selected as model (Fig. 6a). After being intravenously injected with Fe_3_O_4_@C@PMOF for 22 h, fluorescent signal was localized mainly in the liver region than the other organs. The tumor region was lightened slowly and became the brightest tissue of the mice via enhanced permeability and retention (EPR) effect after 26 h. *In vivo T*_*2*_ MR image was also recorded simultaneously (Fig. 6c). The dramatic dimming was observed at tumor area and also demonstrated the high tumor uptake of Fe_3_O_4_@C@PMOF, which was the same as the fluorescence imaging result. To further evaluate the tissue distribution of the nanocomposites, major organs dissected from the mice were harvested and imaged *ex vivo* at 26 h post injection (Fig. 6b). The highest fluorescent intensity of cancer tissue indicated tumor-targeted delivery for imaging, PDT and PTT. The intestine was also lightened and indicated the potential metabolic pathway that Fe_3_O_4_@C@PMOF was excreted through excrement. To further study the excretion of the nanocomposites, high level of Fe was detected in feces of mice after injected with Fe_3_O_4_@C@PMOF (Fig. 6d). Thus, both MR and fluorescent imaging results and *ex vivo* fluorescence images of the tissues confirmed the efficient tumor location of our nanocomposites.

### *In vivo* photothermal and photodynamic synergetic therapy

**Figure 7:**
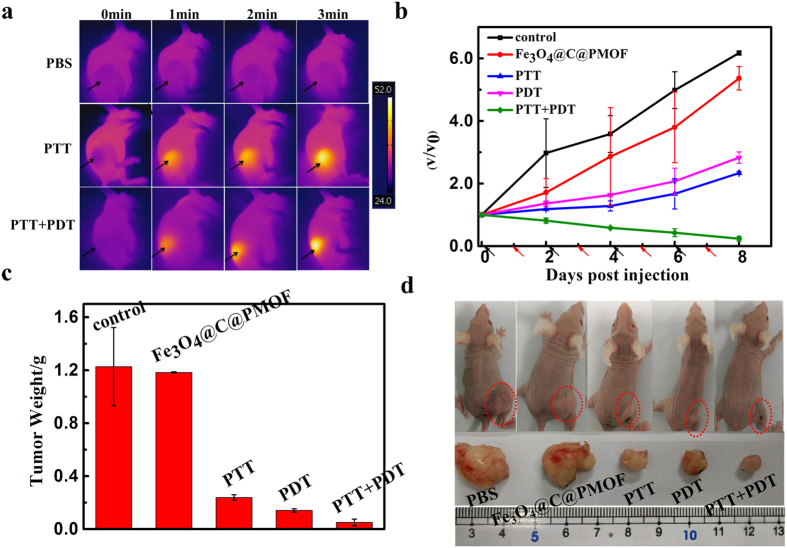
Photothermal and photodynamic synergetic therapy. (**a**) Infrared thermal images of tumor-bearing mice injected with Fe_3_O_4_@C@PMOF (10 mg kg^−1^) or PBS after exposed to 808 nm laser and 808 nm + 655 nm lasers recorded at different time intervals. (**b**) Tumor growth inhibition curve after treatment. V_0_ and V refer to tumor volumes before and after PTT and/or PDT treatment with Fe_3_O_4_@C@PMOF as probe. Black and red arrows refer to injection and irradiation time points, respectively. (**c**) Tumor weight after treatment for eight days. (**d**) Representative photograph of tumor-bearing mice after different treatments. Error bars represent the standard deviations of three mice per group.

Motivated by its high tumor accumulation, Fe_3_O_4_@C@PMOF was used for *in vivo* imaging-guided tumor treatment. Nude mice with subcutaneous MCF-7 breast cancer xenografts were selected as model. For *in vivo* monitoring of the photothermal effect generated from Fe_3_O_4_@C@PMOF, the temperature change of the tumor site was recorded with infrared camera under irradiation of 808 nm laser. To study the *in vivo* synergetic efficiency of PTT and PDT, MCF-7 tumor-bearing mice were randomly divided into five groups. The group injected with saline was regarded as the negative control. All of the other four groups were injected with Fe_3_O_4_@C@PMOF (10 mg kg^−1^). The injected mice without any irradiation were used as the positive control. After the injection for 26 h, the irradiation was carried out. In PTT group, the mice after injection with Fe_3_O_4_@C@PMOF were irradiated with 808 nm laser for 10 min; the mice in PDT group were subjected to the irradiation of 655 nm laser for 10 min. In the PTT-PDT co-therapy group, the mice were firstly irradiated with 808 nm laser for 10 min, followed by the irradiation of 655 nm laser for 10 min. Upon 808 nm laser irradiation, the temperature of the tumor site in the PTT and PTT-PDT co-therapy groups rapidly increased to higher than 50 °C, which is high enough to ablate the cancer cells. For the negative control groups, the tumor tissues didn’t show any significant temperature elevation (Fig. 7a). The injection and irradiation were repeated every two days within 8 days. Tumor sizes and body weights of the mice were monitored every two day after different treatments (Fig. 7b and Supplementary Fig. S11). The size of tumors was normalized to their initial size. Experimental results indicated that the tumor sizes of mice in both negative and positive control groups became larger and larger. In contrast, the tumor growth of the mice after single PTT or PDT inhibited remarkably within 8 days. The PTT-PDT co-therapy group exhibited the highest therapeutic efficacy compared with that of the single PTT or PDT groups. (Fig. 7b and d).

**Figure 8:**
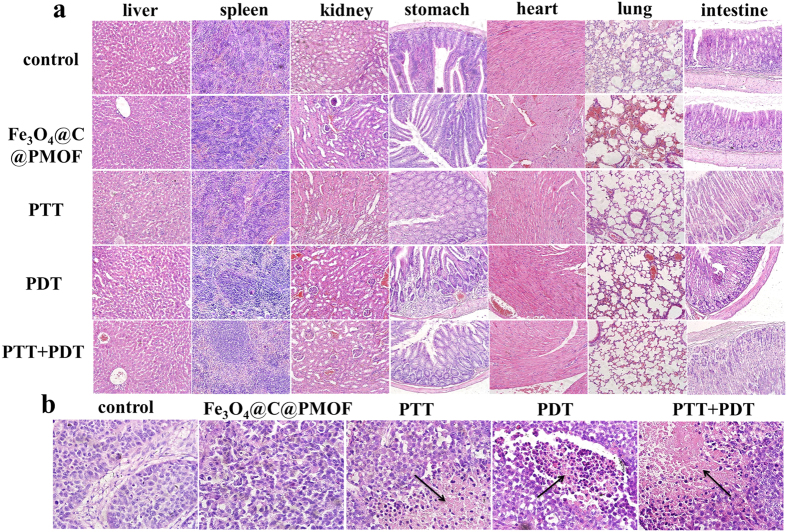
Hematoxylin and eosin (H&E)-stained slices of (**a**) major organs and (**b**) tumor tissue of the mice after 8 days with different treatments. No pathological changes were noticed from the major organs after 8 days after PTT and/or PDT because of the excellent biocompatibility of the nanocomposites. The black arrows refer to necrosis part of tumor. It was obvious that intensive necrosis area was stained by eosin in the dominated tumor section of PTT-PDT treatment group.

Apoptotic and necrotic tumor cells were tested to validate the photo-therapy efficiency (Fig. 8b). Intensive necrosis area was markedly stained by eosin in the dominated tumor section of PTT-PDT treatment group. The results clearly demonstrated that the synergetic therapeutic efficacy of PTT and PDT was superior to any single therapy. The mice after PTT and PDT treatment behaved normally and the weight didn’t decrease remarkably (Supplementary Fig. S11). No pathological changes was noticed for mice after 8 days after PTT and/or PDT, as revealed by hematoxylin and eosin (H&E)-stained major organ slices of the mice because of the excellent biocompatibility of the nanocomposites and the remote controllability, improved selectivity, and safety of phototherapy.

## Discussion

In summary, we developed Fe_3_O_4_@C@PMOF for fluorescence-magnetic resonance dual-modality imaging-guided photothermal and photodynamic cancer dual-therapy by *in situ* growth PMOF shell on Fe_3_O_4_@C core for the first time. The Fe_3_O_4_@C@PMOF nanocomposites featured with some unique advantages over common therapy agents, such as high biocompatibility and stability, and simple self-assembly process to form the MOF shell with the abundant carboxylic groups in the periphery of Fe_3_O_4_@C to interact with Zr^4+^ ions. Effective photothermal and photodynamic therapy of tumors was achieved by passive tumor targeting and excellent photophysical properties of the nanocomposites. Besides, the improved safety and low bio-toxicity of phototherapy, different to chemotherapy, had no damage to normal tissues because of the controllable and local irradiation of the laser. Both irradiation and emission of photo-therapy and fluorescence imaging were around infrared or NIR region, so high penetration depth achieved the efficient photo-therapy and imaging. Fe_3_O_4_@C@PMOF with low biotoxicity shows promise for future clinical translation as validated by the dual-modality imaging-guiding synergetic therapy. The results demonstrate the availability of as-synthesized MCTPs on tumor and illustrate great potential in tumor diagnosis and treatment.

## Methods

### Animal experiments

To validate the dual-modality imaging-guided photothermal and photodynamic dual-therapy, we used female BALB/c-nu mice with the body weight of 18–22 g as model. The mice were obtained from the Institute of Hematology & Hospital of Blood Disease, Chinese Academy of Medical Sciences & Peking Union Medical College with the license No. SCXK-2014-0013, Tianjin, China. The mice were housed one per cage in a specific pathogen-free environment and had free access to standard solid pellet food (HFK, Beijing, China) and water. We confirmed that all experimental protocols were approved by the Institutional Animal Care Committee of Nankai University and all methods were carried out in accordance with the relevant guidelines and regulations from the Institutional Animal Care Committee of Nankai University.

### Synthesis of Fe_3_O_4_@C nanospheres

Fe_3_O_4_@C micro-structure was fabricated by one-pot solvothermal strategy according to previous report^31^. Briefly, 0.08 g of ferrocene was dissolved in 32 mL acetone. 0.4 mL of 30% hydrogen peroxide was added and the mixture solution was transferred to a 50 mL Teflon-lined stainless autoclave. The mixture was then kept at 210 °C for 24 h. After the autoclave was cooled to room temperature, the products were collected by a magnet after ultrasonication for 20 min. Black solid was washed with acetone and ethanol three times and dried in vacuum at 40 °C for 24 h to obtain Fe_3_O_4_@C nanospheres.

### Preparation of Fe_3_O_4_@C@PMOF

1 mL of ZrCl_4_ dimethyl formamide (DMF) solution (2.6 mmol L^−1^) was added to the suspension of 10 mg Fe_3_O_4_@C nanospheres in 5 mL DMF. The mixture was transferred to a 25 mL three-necked flask and kept at 120 °C with vigorous stirring for 30 min. Then, 100 μL of TCPP DMF solution (38 μmol L^−1^) was added drop-wise into the mixture in 2 min. The reaction proceeded for another 3 h at 120 °C. The product was collected magnetically and washed with DMF and ethanol and dried for further use.

### *In vitro* singlet oxygen generation

The singlet oxygen (^1^O_2_) generation capacity of Fe_3_O_4_@C@PMOF was tested using 9, 10-anthracenediyl-bis (methylene) dimalonic acid (ABDA) method after laser irradiation. ^1^O_2_ generated from Fe_3_O_4_@C@PMOF oxidizes ABDA to decrease its UV absorption. PBS (pH 7.4) containing 20 μmol L^−1^ Fe_3_O_4_@C@PMOF and 200 μmol L^−1^ ABDA was therefore irradiation with 655 nm laser (0.3 W cm^−2^). The absorbance spectra of solution were recorded at different time points. The stability of ABDA to PBS, light, and single Fe_3_O_4_@C@PMOF was also tested as control.

### Cytotoxicity and photoxicity of Fe_3_O_4_@C@PMOF

The cytotoxicity and photoxicity of Fe_3_O_4_@C@PMOF were evaluated by the viability of MCF-7 cells using a standard methyl thiazolyl tetrazolium (MTT) assay. Four groups were tested separately with different treatments. Briefly, the cells were incubated to 96-well culture plates at a density of 5 × 10^3^ cells per well in culture medium. Fe_3_O_4_@C@PMOF was introduced to the medium at the concentration between 0 and 400 μg mL^−1^ and incubated for 8 h after MCF-7 cells reached 90–95% confluences. Then, the PDT and PTT groups were under 655 nm for 10 min (0.3 W cm^−2^) and 808 nm irradiation for 10 min (1.0 W cm^−2^), respectively. The PDT-PTT dual-therapy group was firstly under 808 nm irradiation for 10 min and then 655 nm for 10 min. N, N’-dimethyl sulfoxide (150 μL) was used to completely liberate the formazan crystals. The absorbance at 490 nm was measured to calculate the cell viability.

### *In vitro* MR test with Fe_3_O_4_@C@PMOF as probe

In *vitro* MR imaging with Fe_3_O_4_@C@PMOF as probe was carried out at different Fe concentrations (0.07, 0.14, 0.28, 0.56, 1.12 mM) with a 1.2 T MR Imaging System, Huantong, Shanghai, China. The *T*_*2*_ value could be tested directly with the 1.2 T MR imaging system, and Fe content of Fe_3_O_4_@C@PMOF was determined by inductively coupled plasma-atomic emission spectroscopy. The slope of the linear fitting equation between 1/*T*_*2*_ and Fe content was the *r*_*2*_ value. Images were recorded using a 50 mm animal coil and a 2D gradient imaging sequence. The MR parameters were described as follows: spin-echo *T*_*2*_-weighted MR sequence, TR/TE = 5000/64.6 ms, FOV = 50 × 80 mm^2^, matrix = 512 × 256, slice thickness = 0.4 mm, 30.0 °C.

### *In vivo* MR imaging

*In vivo* MR imaging was performed on the mice or MCF-7 tumor-bearing mice anesthetized with 4% chloral hydrate (6 mL kg^−1^). After intravenous injection of Fe_3_O_4_@C@PMOF solution (20 mg kg^−1^) into the mice, the MR images were recorded by positioning the mice on the animal plate of imaging system. The MR imaging was recorded on a 1.2 T MR imaging system, Huantong, Shanghai, China. Images were obtained using a small animal coil, before and at subsequent intervals following injection with the imaging sequence: TR/TE = 300/32.6 ms; FOV = 50 mm × 80 mm; matrix = 512 × 256; slice thickness = 0.4 mm without gap; 128 coronal or axial slices. To study the contents of nanocomposites in excretion of mice, mice after intravenous injection with Fe_3_O_4_@C@PMOF were housed in metabolic cages to collect their urine and feces. The collected urine and feces were digested by chloroazotic acid and measured by ICP-AES.

### *In vivo* fluorescence imaging

*In vivo* fluorescence images of nude mice and tumor-bearing mice were recorded after anesthetized with 4% chloral hydrate (6 mL kg^−1^). After intravenous injection of Fe_3_O_4_@C@PMOF solution (20 mg kg^−1^) into the mice, the fluorescence images were recorded with NightOWL LB 983 small animal *in vivo* imaging system (Berthold Technologies GmbH & Co. KG, Germany) with the optimal wavelength of PMOF (Ex = 550 nm, Em = 660 nm). The data were treated with IndiGO software.

### PTT-PDT dual-therapy of tumor-bearing mice with Fe_3_O_4_@C@PMOF as probe

The efficiency of PTT-PDT dual-therapy of Fe_3_O_4_@C@PMOF was tested with tumor-bearing mice (18−22 g, n = 3 per group) as model after anesthetized with 4% chloral hydrate (6 ml kg^−1^). The mice were separated into five groups. The mice injected intravenously with saline were used as negative control, while the mice injected with Fe_3_O_4_@C@PMOF (10 mg kg^−1^) without any laser irradiation were used as positive control. The other mice injected with Fe_3_O_4_@C@PMOF were subjected to laser irradiation for PTT, PDT, and PTT-PDT dual-therapy, respectively. The distribution of Fe_3_O_4_@C@PMOF was monitored with fluorescent imaging. When the signal of Fe_3_O_4_@C@PMOF reached zenith at the tumor site, the tumor site of the mice in PDT group were irradiated with 655 nm laser for 10 min (0.3 W cm^−2^), while the mice in PTT group were irradiated under 808 nm laser for 10 min (1.0 W cm^−2^). The mice in PTT-PDT dual-therapy group were firstly irradiated under 808 nm laser for 10 min (1.0 W cm^−2^) and then 655 nm laser (0.3 W cm^−2^) for 10 min, respectively. The weights and tumor sizes of the mice were monitored simultaneously. The tumor volumes were calculated as (width^2^ × length)/2 based on the previous report^28^. The main organs of mice were collected after treatment for 8 days. Hematoxylin and eosin (H&E) stained images were used to investigate the biotoxicity. The body weights of the mice were assessed with a counter balance within 8 days.

## Additional Information

**How to cite this article**: Zhang, H. *et al*. Fluorescence and Magnetic Resonance Dual-Modality Imaging-Guided Photothermal and Photodynamic Dual-Therapy with Magnetic Porphyrin-Metal Organic Framework Nanocomposites. *Sci. Rep.* **7**, 44153; doi: 10.1038/srep44153 (2017).

**Publisher's note:** Springer Nature remains neutral with regard to jurisdictional claims in published maps and institutional affiliations.

## Supplementary Material

Supplementary Information
