# Supplementary material for: Fluorescence and Magnetic Resonance Dual-Modality Imaging-Guided Photothermal and Photodynamic Dual-Therapy with Magnetic Porphyrin-Metal Organic Framework Nanocomposites
Source: Sci Rep. 2017 Mar 8;7:44153. doi: 10.1038/srep44153 (PMC5341151; doi:10.1038/srep44153)
Supplement: Supplementary Information [file srep44153-s1.docx]

**Fluorescence and Magnetic Resonance Dual-Modality Imaging-Guided Photothermal and Photodynamic Dual-Therapy with Magnetic Porphyrin-Metal Organic Framework Nanocomposites**

Hui Zhang^1^, Yu-Hao Li^2^, Yang Chen^2^, Man-Man Wang^3^, Xue-Sheng Wang^3^, Xue-Bo Yin*^1,4^

^1^ State Key Laboratory of Medicinal Chemical Biology and Tianjin Key Laboratory of Biosensing and Molecular Recognition, College of Chemistry, Nankai University, Tianjin 300071, China

^2^ Tianjin Key Laboratory of Tumor Microenviroment and Neurovascular Regulation, School of Medicine, Nankai University, Tianjin 300071, China

^3^ School of Public Health, North China University of Science and Technology, Tangshan 063000, Hebei, China

^4^ Collaborative Innovation Center of Chemical Science and Engineering (Tianjin), Nankai University, Tianjin 300071, China

* E-mail: xbyin@nankai.edu.cn; Fax: (+86) 022-23503034

1. **Experimental section**

**1.1 Materials**

Ferrocene (Fe(C_5_H_5_)_2_, 99%) was obtained from Alfa-Asia, Tianjin, China. Hydrogen peroxide (H_2_O_2_, 30%) was from Aladdin-Asia, Shanghai, China. 5, 10, 15, 20–Tetrakis (4-carboxyl)-21H, 23H-porphine (TCPP) was purchased from TCI Chemical Ind. Develop Co., Shanghai. China. ZrCl_4_ was obtained from Alfa-Asia, Tianjin, China. Dimethylformamide (DMF) and other solvents were from Concord Reagent Co, Tianjin, China. 9, 10-anthracenediyl-bis (methylene) dimalonic acid (ABDA) was purchased from Aladdin biotechnology Co, shanghai, china. All reactants were reagent grade and were used as purchased without further purification. Ultra-pure water was prepared with an Aquapro system (18.25 MΩ).

**1.2 Instrumentation and characterization**

UV-Vis absorption spectrum was recorded by a UV-2450-visible spectrophotometer (Shimadzu, Japan). The steady-state ﬂuorescence experiments were performed on a FL-4600 Fluorescence Spectrometer (Hitachi, Japan) equipped with a plotter unit and a quartz cell (1 cm×1 cm). The slit width was 10 nm for both excitation (550 nm) and emission. Infrared spectra were obtained by Bruker TENSOR 27 Fourier transform infrared spectroscopy. Thermogravimetric analysis (TGA) was performed on a PTC-10ATG-DTA analyzer heated from 20°C at a ramp rate of 10 °C min^-1^ under air.

Transmission electron microscopy (TEM) images were recorded with Tecnai G2 F20, FEI Co., America operated at an accelerating voltage of 200 kV. Scanning electron microscopy (SEM) images were recorded with JSM-7500F, Japan. XRD patterns were obtained by a D/max-2500 diffractometer (Rigaku, Japan) using Cu-Kα radiation (λ=1.5418 Å). The content of Fe and Zr was measured by Inductively Coupled Plasma Atomic Emission Spectroscopy (ICP-AES), IRIS advantage, Thermo, USA. DLS size distribution and zeta potential were recorded with Zetasizer Nano ZS, Malvern, England.

**2. Results and discussions**


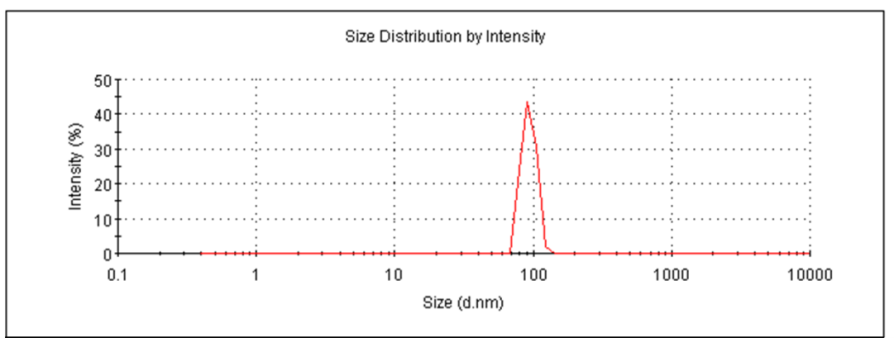


Figure S1 | DLS measured size distribution of Fe_3_O_4_@C@PMOF. The average size of Fe_3_O_4_@C@PMOF was 98 nm.

**
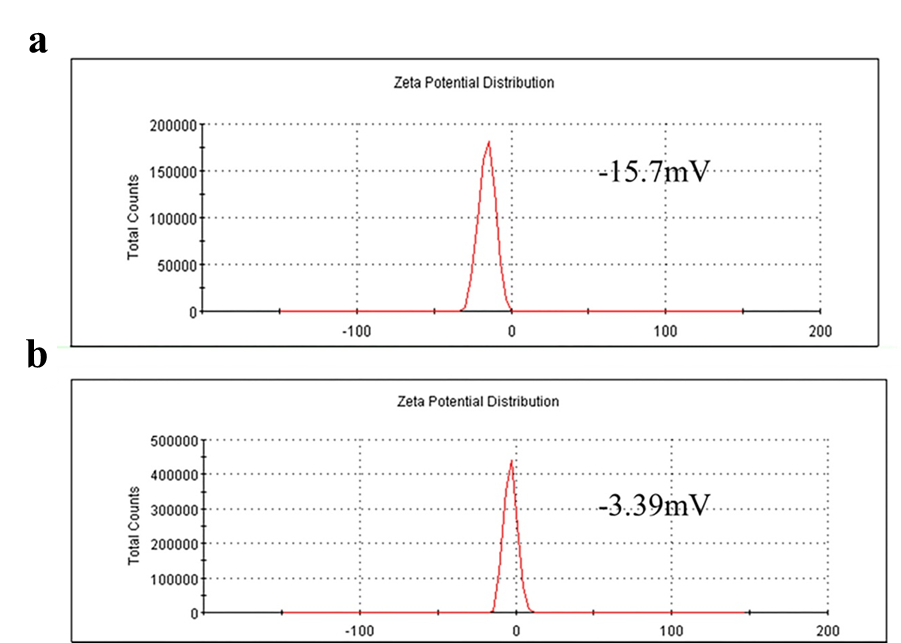
**

Figure S2 | Zeta potential of (a) Fe_3_O_4_@C and (b) Fe_3_O_4_@C@PMOF nanocomposites. -15.7 and -3.39 mV of zeta potential was observed from the Fe_3_O_4_@C and Fe_3_O_4_@C@PMOF, respectively.


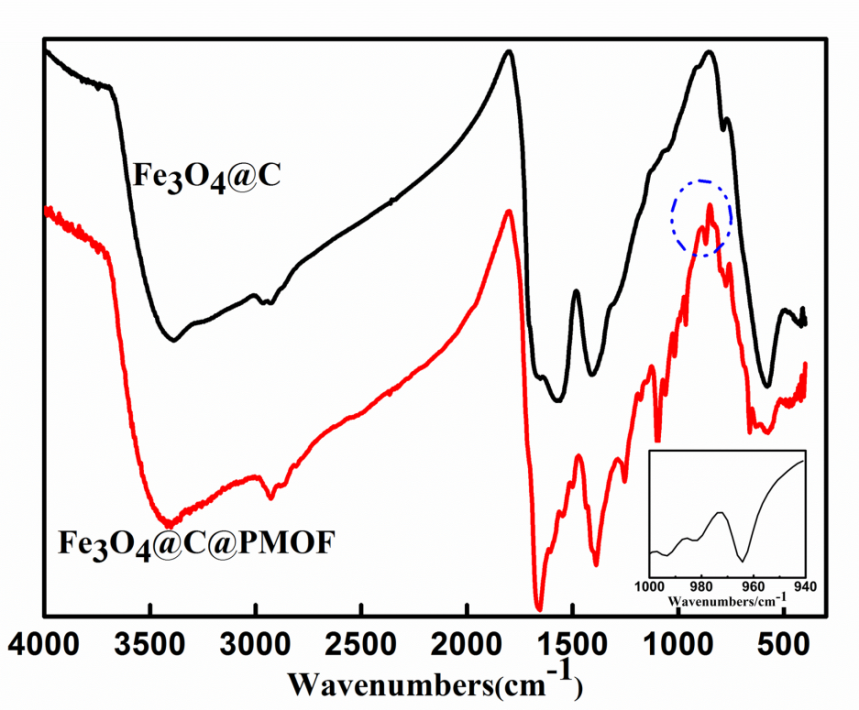


Figure S3 | FT-IR spectra of Fe_3_O_4_@C and Fe_3_O_4_@C@PMOF nanocomposites. The amplification part around wavenumber 964 was added in the inset. The comparison between the FT-IR spectra of Fe_3_O_4_@C and Fe_3_O_4_@C@PMOF validated the successful formation of PMOF shell on the surface of Fe_3_O_4_@C nanoparticles.





**Figure S4** **|** **Thermogravimetric analysis curves of Fe_3_O_4_@C and Fe_3_O_4_@C@PMOF.** The gradual weight loss before 200 °C was attributed to the removal of solvents, including acetone and DMF, from both Fe_3_O_4_@C and Fe_3_O_4_@C@PMOF. And the removal of carbon shell of Fe_3_O_4_@C was at around 300℃.The large weight loss of Fe_3_O_4_@C@PMOF occurred at around 400℃ was assigned to the collapse of the PMOF skeleton upon the decomposition of TCPP.


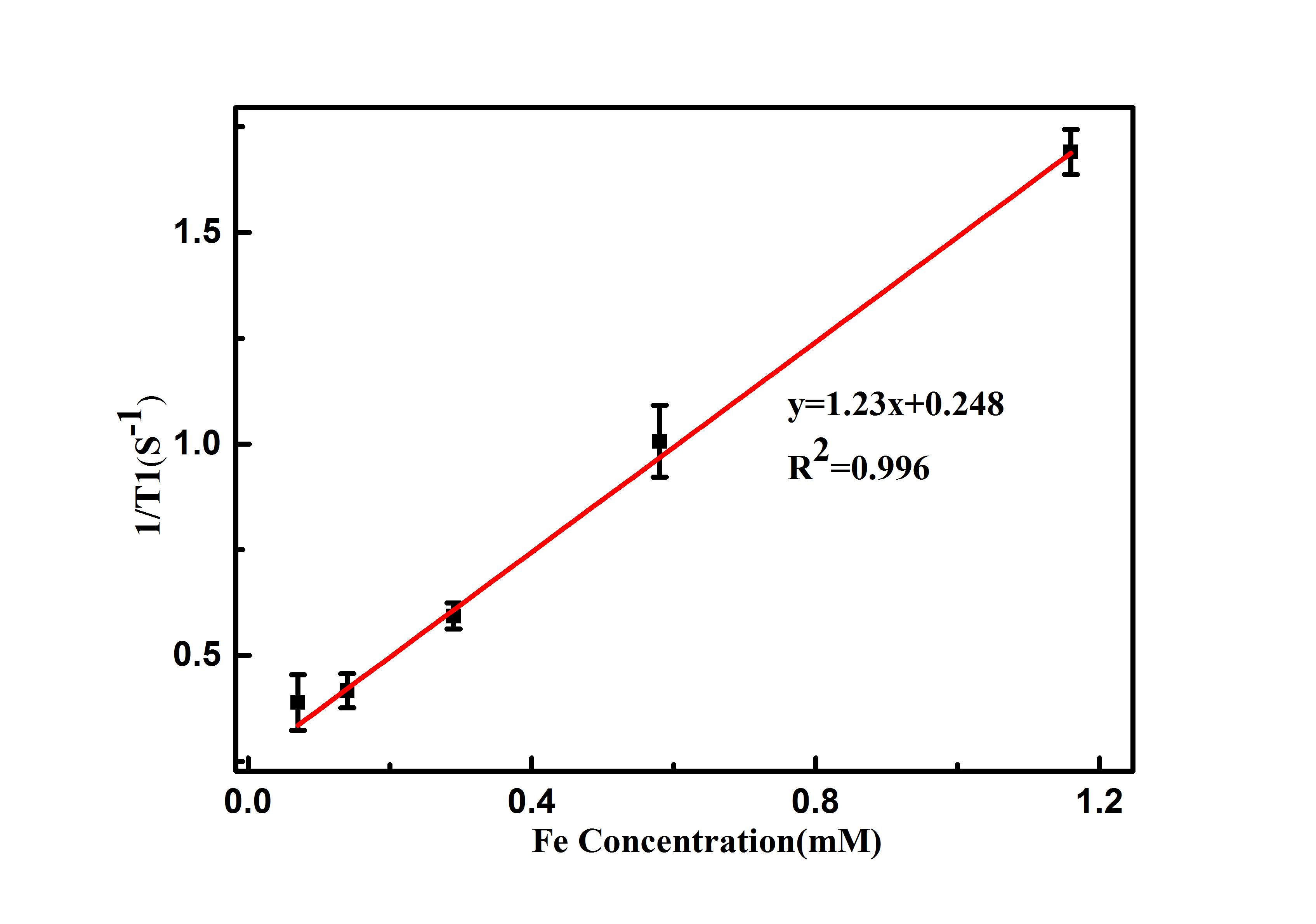


Figure S5 | The *r_1_* relaxivity curves of Fe_3_O_4_@C@PMOF. Fe_3_O_4_@C@PMOF as probe was carried out at different Fe concentrations (0.07, 0.14, 0.28, 0.56, 1.12 mM) with a 1.2 T MR imaging system. *T_1_* value could be tested directly with the 1.2 T MR imaging system and Fe content of Fe_3_O_4_@C@PMOF was determined with inductively coupled plasma-atomic emission spectroscopy. The slope of the linear fitting equation between 1/*T_1_* and Fe content was the *r_1_* value. The *r_2_*/*r_1_* ratio was 59.0, and therefore Fe_3_O_4_@C@PMOF showed the potential for *T_2_*-weighted MR imaging.

**
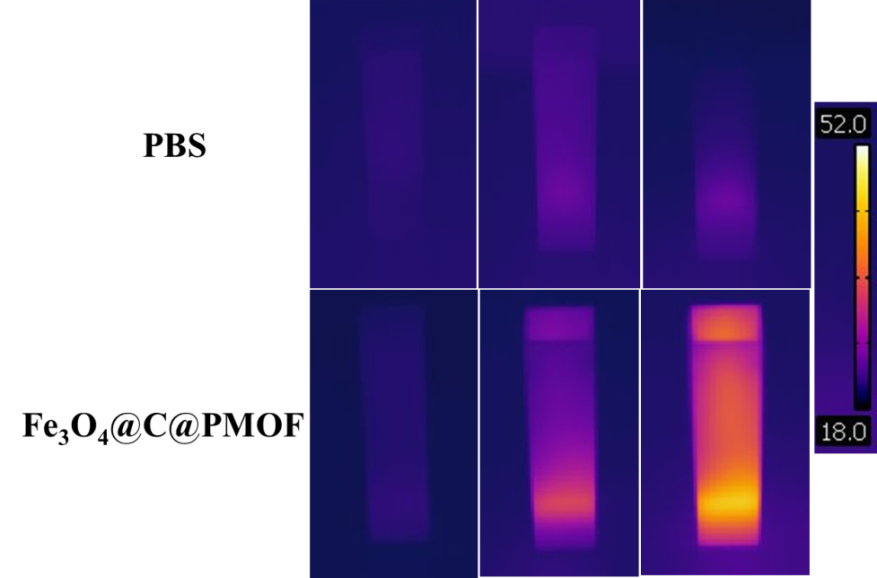
**

Figure S6 | Infrared thermal photograph of PBS and Fe_3_O_4_@C@PMOF solution. The solution was at 808 nm irradiation (1.0 W cm^-2^) for 0, 2, and 5 min from left to right.


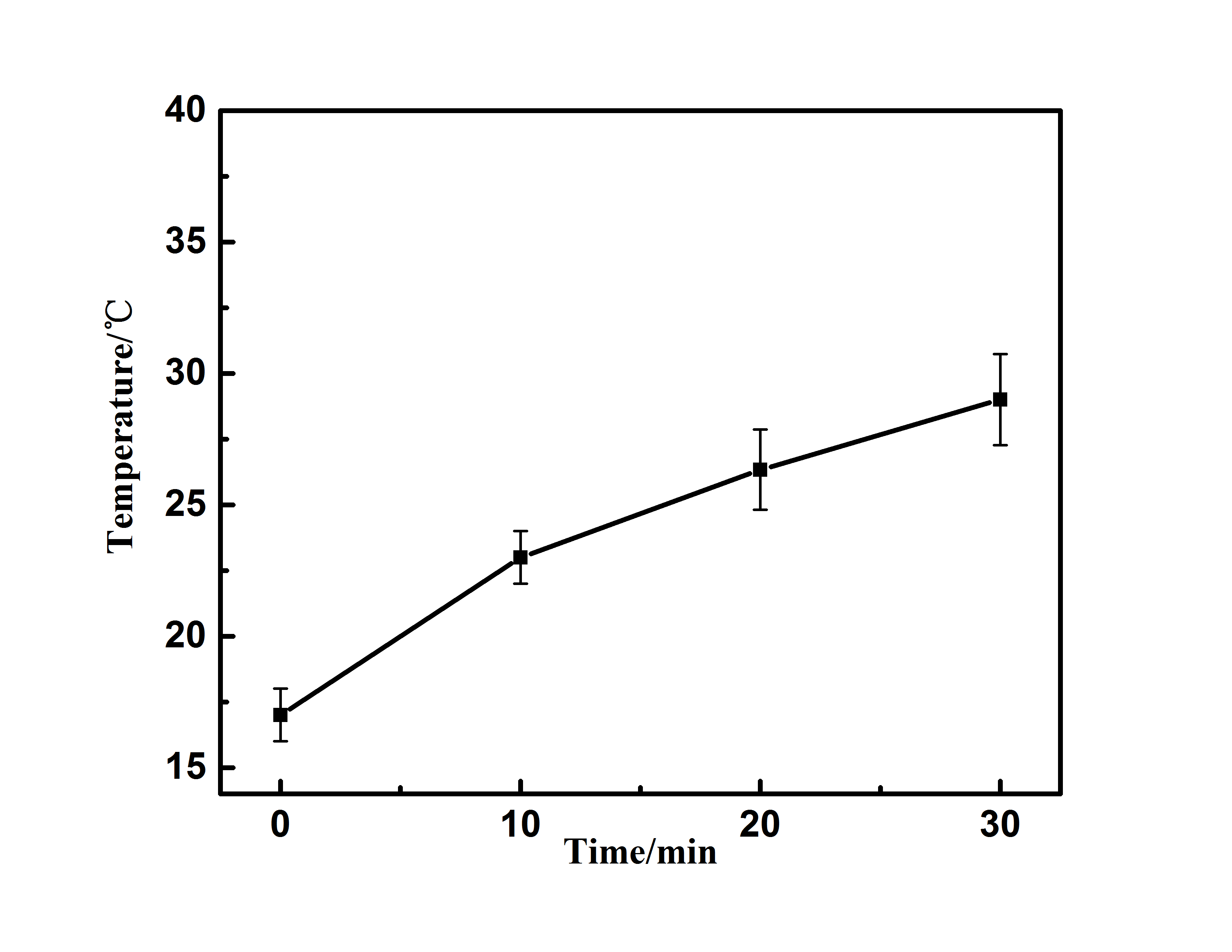


Figure S7 | Temperature elevation of Fe_3_O_4_@C@PMOF as a function of time upon exposure to 655 nm laser at 0.3W cm^-2^ for 30 min. The temperature gave rise to 29 °C, which was negligible for cancer cells.

**

**

Figure S8 | Stability of ABDA against time, light, and Fe_3_O_4_@C@PMOFs. It was quite obvious that ABDA absorbance was stable for single Fe_3_O_4_@C@PMOF or light.


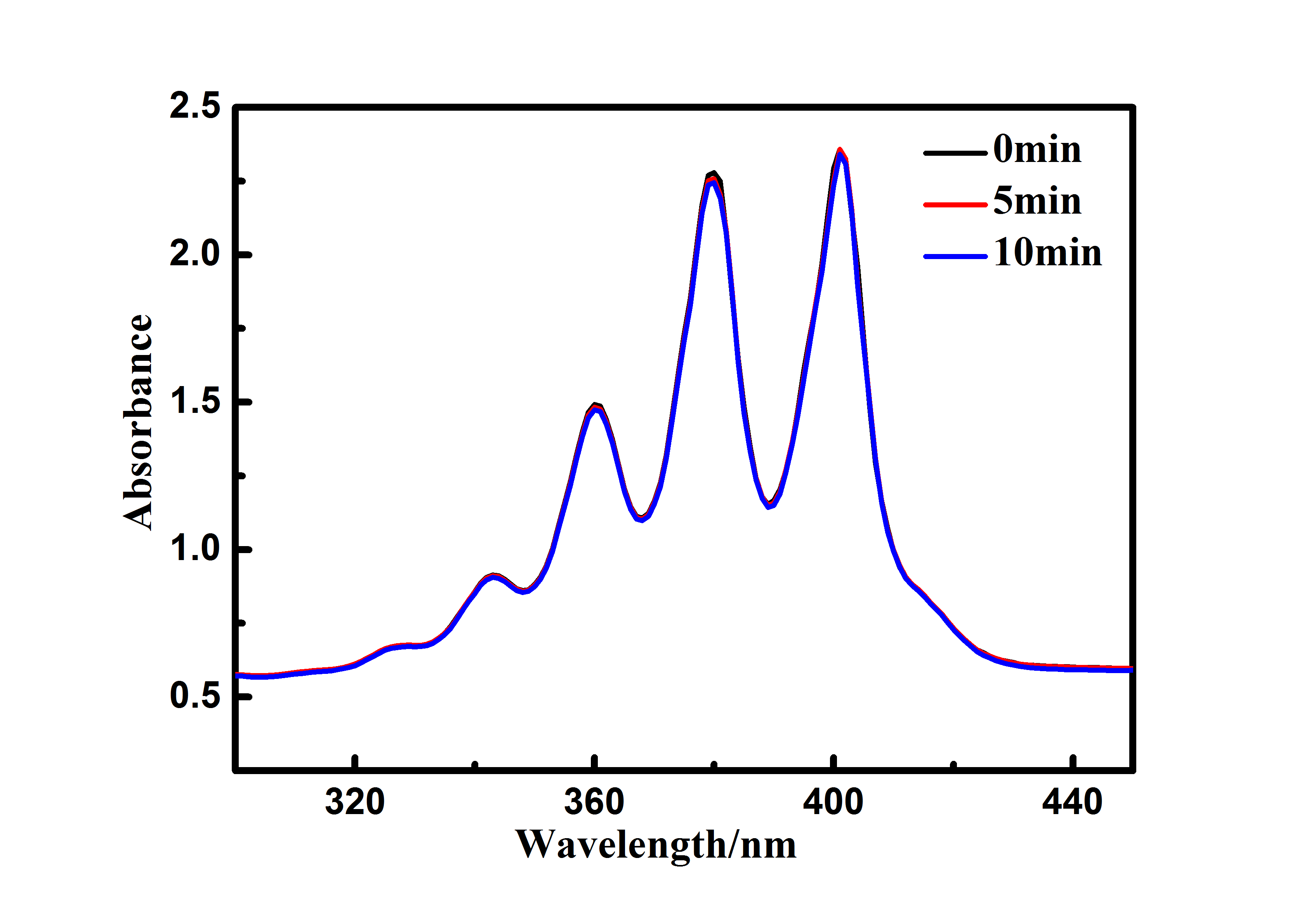


Figure S9 | Absorbance spectra of ABDA (200 μmol L^-1^) in the presence of Fe_3_O_4_@C@PMOF nanocomposites (20 μmol L^-1^) over diﬀerent periods of time under irradiation of 808 nm. The ABDA absorption at 379 nm was stable under 808 nm laser irradiation.


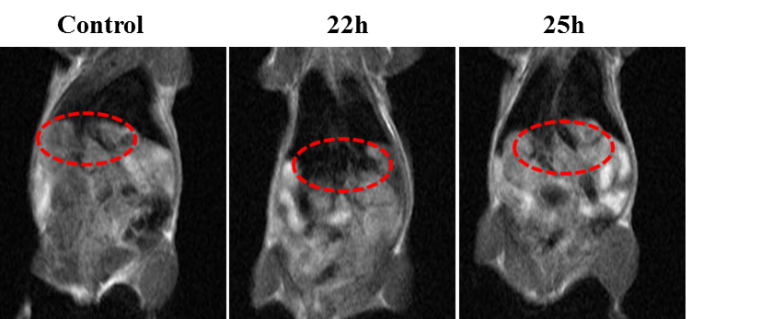


Figure S10 | MR imaging of nude mice before and after intravenous injection of Fe_3_O_4_@C@PMOF. The injected dose of Fe_3_O_4_@C@PMOF was 20 mg kg^-1^. The red dot lines refer to liver region.





**Figure S11 | Weight trends of the tumor-bearing mice after different treatment.** The weight of the mice after PTT or/and PDT treatment didn’t decrease remarkably. Error bars represent the standard deviations of 3 mice per group.
